# Hepatitis B: changes in epidemiological features of Afro-descendant communities in Central Brazil

**DOI:** 10.1038/s41598-020-63094-5

**Published:** 2020-04-21

**Authors:** Livia Alves Lima, Bárbara Vieira do Lago, Sabrina Moreira dos Santos Weis-Torres, Regina Maria Bringel Martins, Gabriela Alves Cesar, Larissa Melo Bandeira, Grazielli Rocha Rezende, Andrea de Siqueira Campos Lindenberg, Selma Andrade Gomes, Ana Rita Coimbra Motta-Castro

**Affiliations:** 1 Federal University of Mato Grosso do Sul, Campo Grande, MS Brazil; 2 Oswaldo Cruz Foundation, FIOCRUZ, Rio de Janeiro, RJ Brazil; 3 Institute of Technology in Immunobiology, Bio-Manguinhos, FIOCRUZ, Rio de Janeiro, RJ Brazil; 4 Federal University of Goiás, Goiânia, GO Brazil; 5 Oswaldo Cruz Foundation, FIOCRUZ Mato Grosso do Sul, Ministry of Health, Campo Grande, MS Brazil

**Keywords:** Infectious-disease diagnostics, Hepatology, Risk factors

## Abstract

Hepatitis B virus (HBV) infection is still a concern in vulnerable populations. In a study performed by our team in 1999–2003 in two Afro-Brazilian communities, Furnas dos Dionísios (FD) and São Benedito (SB), high prevalence rates of HBV exposure (42.7% and 16.0%, respectively), high susceptibility to HBV (55.3% and 63.0%) and low HBV vaccination like profile rates (2.0% and 21.0%) were observed. In 2015–2016, we reassessed HBV epidemiological and molecular features in these two communities to verify the impact of health actions adopted in the last years. The prevalence rate of HBV exposure among the enrolled 331 subjects was 35.3% in FD and 21.8% in SB. HBV chronic infection (5.8% in FD, 4.9% in SB) remained high. The rate of HBV vaccination like profile increased from 10.7% to 43.5% (2.0% to 45.9% in FD, 21.0% to 39.5% in SB) while susceptible subjects declined from 58.9% to 26.3% (55.3% to 18.8% in FD, 63.0% to 38.7% in SB). Among 18 HBsAg positive samples, 13 were successfully sequenced (pre-S/S region). Phylogenetic analyses showed that all isolates belong to HBV subgenotype A1, clustering within the Asian-American clade. Despite the maintenance of high prevalence rate of HBV exposure over these 13 years of surveillance, significant improvements were observed, reinforcing the importance of facilitated HBV vaccination to difficult-to-access population to close gaps in prevention.

## Introduction

Hepatitis B virus (HBV) infection is still a major public health issue worldwide despite the availability of an effective vaccine and potent antiviral treatments^1–3^. An estimated 2 billion people have been infected with HBV and 257 million people are chronic carriers of the virus. Serious complications of chronic HBV infection such as cirrhosis, liver failure, and hepatocellular carcinoma accounted for 887 000 deaths in 2015^4,5^.

Hepatitis B surface antigen (HBsAg) is the main HBV clinical marker indicating acute or chronic infection and its prevalence is used to categorize HBV endemicity as low, intermediate low, intermediate high, or high^2,6^. Even though Brazil is now a low endemic country, it has some areas with high HBV prevalence, especially in the Amazon basin, some counties of Southern Brazil and, isolated communities^7–9^.

In Brazil, HBV immunization was first implemented in 1989 for infants in the western area of the Amazonas State and since 1998 HBV vaccine has been incorporated into the immunization schedule for infants as a national policy^10^. Nowadays, HBV vaccination is available to all individuals regardless of age or risk group, being considered satisfactory and effective, moving Brazil from moderate HBV endemicity to low-endemicity classification^7^.

Although Brazilian multi-center population-based studies have observed a decrease in HBV infection prevalence in the last decades, especially among children and young adults^10,11^, vaccination is still a challenge in difficult-to-access populations and therefore, observations cannot be extrapolated to rural or vulnerable populations^7^.

Brazil is an ethnically mixed country, composed of descendants of European colonizers, native Amerindians and African slaves. Roughly five million African captives were brought to Brazil during centuries of slavery. Some escaped from forced work, setting in remote valleys. These runaway-slave descendants remained in isolated communities, called “Quilombos,” without significant additional admixture since their establishment^12^. Remnant quilombo communities can still be found in almost all macro-regions of Brazil^13^.

Between 1999 and 2003, we found high prevalence rates of HBV exposure in the two largest remnant quilombo communities from Central Brazil, named Furnas dos Dionísios (FD) and São Benedito (SB) (42.7% and 16.0%, respectively). Additionally, we observed high rates of susceptible individuals (55.3% and 63.0%) and also a low proportion (2.0% and 21.0%) of individuals presenting serological HBV vaccination like profile, i.e. isolate anti-HBs ≥10mIU/mL^12,14–16^. Based on these alarming findings, in 2003, our research group conducted actions for health promotion such as HBV vaccine supply, health educational campaigns, and referral of active hepatitis B cases for public clinical health care. These health actions were promoted in partnership with the Public Health authorities of the State of Mato Grosso do Sul in response to the high prevalence rate of HBV exposure and hepatitis B chronic infection in these communities^15^. Given the above, we reassessed the hepatitis B epidemiological profile in these two Afro-Brazilian communities over 13 years after the first study, in order to verify possible changes in HBV epidemiological features and the impact of the measures adopted in the past with a view to future interventions.

## Results

### Baseline sociodemographic and behavioral characteristics of the studied population

The socio-demographic and behavioral characteristics among participants according to HBV serological profile are shown in Table 1. A total of 331 Afro-descendants agreed to participate in the study, most of them were females (57.4%) and were from the Furnas do Dionisio community (62.5%). The age of the individuals ranged from 1 to 89 years (mean ± SD: 35 ± 21). Most of the participants (74.9%) reported having sexual activity and 81.5% of those, also reported irregular condom use. In addition, 28.7% reported having a family member infected with hepatitis B and 36.9% reported sharing personal hygiene items, such as razor blade, toothbrush and nail/cuticle pliers. Regarding HBV vaccination, 60.4% of the participants self-reported at least one shot of HBV vaccine.

**Table 1:** Hepatitis B virus infection (current and exposed), HBV vaccination like profile, and susceptibility by demographic, behavioral, and clinical characteristics among Afro-descendants in Central Brazil, 2015–2016.

Variables	Total no. (%)	Current infected no. (%)	Exposed no. (%)	Vaccination like profile no. (%)	Susceptible no. (%)
**Total**	331 (100)	18 (5.4)	82 (24.8)	144 (43.5)	87 (26.3)
**Community**					
SB	124 (37.5)	6 (4.9)	21 (16.9)	49 (39.5)	48 (38.7)
FD	207 (62.5)	12 (5.8)	61 (29.5)	95 (45.9)	39 (18.8)
**Gender**					
Female	190 (57.4)	4 (2.1)	46 (24.2)	94 (49.5)	46 (24.2)
Male	141 (42.6)	14 (9.9)	36 (25.5)	50 (35.5)	41 (29.1)
**Age (years)**					
1–10	43 (13.0)	0 (0.0)	1 (2.3)	26 (60.5)	16 (37.2)
11–20	58 (17.5)	2 (3.4)	4 (6.9)	36 (62.1)	16 (27.6)
21–30	51 (15.4)	4 (7.8)	4 (7.8)	40 (78.5)	3 (5.9)
31–40	48 (14.5)	5 (10.4)	11 (22.9)	16 (33.3)	16 (33.3)
41–50	51 (15.4)	2 (3.9)	25 (49.0)	8 (15.7)	16 (31.4)
51–60	43 (13.0)	1 (2.3)	18 (41.9)	12 (27.9)	12 (27.9)
> 60	37 (11.2)	4 (10.8)	19 (51.4)	6 (16.2)	8 (21.6)
**Educational level (years)**					
≥ 10	81 (24.5)	8 (9.9)	10 (12.3)	48 (59.3)	15 (18.5)
1–9	224 (67.7)	9 (4.0)	63 (28.1)	86 (38.4)	66 (29.5)
0	26 (7.8)	1 (3.9)	9 (34.6)	10 (38.4)	6 (23.1)
**Monthly income**^**a**^					
<1 minimum wages	101 (30.6)	2 (1.9)	24 (23.8)	51 (50.5)	24 (23.8)
1–3 minimum wages	211 (63.9)	16 (7.6)	52 (24.6)	90 (42.7)	53 (25.1)
>3 minimum wages	18 (5.5)	0 (0.0)	6 (33.3)	3 (16.7)	9 (50.0)
**History of a family member HBV-infected**^**a**^					
No	216 (69.5)	5 (2.3)	44 (20.4)	105 (48.6)	62 (28.7)
Yes	95 (30.5)	12 (12.6)	29 (30.6)	31 (32.6)	23 (24.2)
**STI history**^**b**^					
No	219 (90.1)	17 (7.8)	64 (29.2)	84 (38.4)	54 (24.6)
Yes	24 (9.9)	1 (4.2)	7 (29.2)	10 (41.6)	6 (25.0)
**Self-reported hepatitis B vaccination**^**a**^					
Not vaccinated	64 (24.2)	2 (3.1)	17 (26.6)	25 (39.0)	20 (31.3)
Vaccinated	200 (75.8)	13 (6.5)	50 (25.0)	93 (46.5)	44 (22.0)

^a^The total represents the number of individuals who answered the question. ^b^The total represents the number of individuals ≥13 years old who answered the question.

### HBV serological profile

Of the 331 individuals, 18 (5.4%) were positive for HBsAg and total anti-HBc, five (1.5%) were total anti-HBc only, and 77 (23.3%) had anti-HBs and total anti-HBc markers, resulting in an HBV exposure rate of 30.2% (95% CI: 25.5–35.4) (Table 2). All 18 HBsAg-positive individuals were anti-HBc IgM negative, 17 of them were HBeAg negative/anti-HBe positive and one was HBeAg positive/anti-HBe undetermined. The FD community had a higher (35.3%) prevalence of HBV exposure when compared to SB community (21.8%; p = 0.010), whereas SB community had a higher proportion of individuals susceptible to HBV infection (18.8% vs. 38.7%; p < 0.001) (Table 2).

**Table 2:** Prevalence of hepatitis B virus serological markers among Afro-descendants in Central Brazil, 2015–2016.

Category	Serological marker	All Afro-descendants		Furnas dos Dionísios		São Benedito		p-value
		(N = 331)		(N = 207)		(N = 124)		
		**n (%)**	**95% CI¹**	**n (%)**	**95% CI¹**	**n (%)**	**95% CI¹**	
HBV exposure								
	HBsAg/anti-HBc	18 (5.4)	3.4–8.5	12 (5.8)	3.3–10.0	6 (4.9)	2.2–10.5	0.710
	Alone Anti-HBc	5 (1.5)	0.6–3.6	1 (0.5)	0.0–3.4	4 (3.2)	1.2–8.4	0.048*
	Anti-HBc/anti-HBs	77 (23.3)	19.0–28.1	60 (29.0)	23.2–35.6	17 (13.7)	8.6–21.1	0.001*
	Any HBV infection marker	100 (30.2)	25.5–35.4	73 (35.3)	29.0–42.1	27 (21.8)	15.3–30.0	0.010*
Vaccination like profile	Alone anti-HBs	144 (43.5)	38.2–48.9	95 (45.9)	39.2–52.8	49 (39.5)	31.2–48.5	0.257
Not exposed, susceptible	Absence of marker	87 (26.3)	21.8–31.3	39 (18.8)	14.0–24.8	48 (38.7)	30.5–47.7	<0.001*

(¹) Confidence Interval.

**Figure 1:**
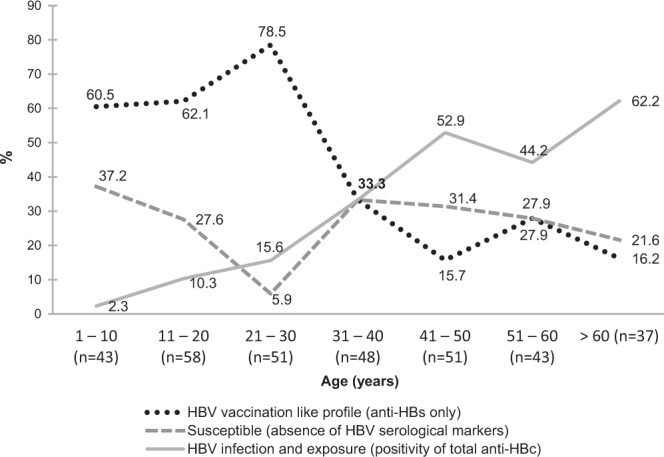
HBV serological profile among Afro-descendants according to age (years), Brazil Central, 2015–2016 (n = 331).

**Figure 2:**
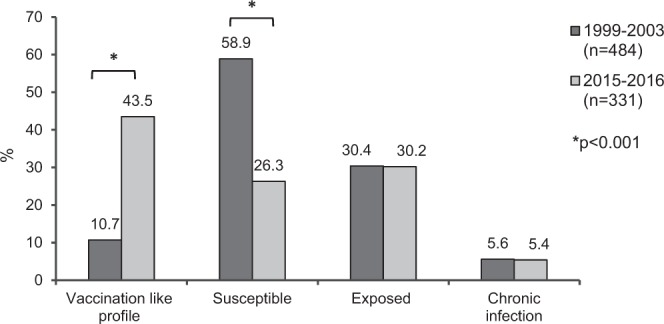
Comparison between HBV epidemiological features among Afro-descendants in the past (1999–2003) and present (2015–2016), Brazil Central.

As shown in Fig. 1, the group aged >60 years had the highest prevalence of HBV exposure (62.2%), and the group aged 21–30 years presented the highest prevalence of anti-HBs alone (78.5%). After multiple linear regression analysis, being a FD community resident, increasing age and history of family member HBV-infected remained associated with HBV exposure after adjustment for potential factors associated with this infection (Table 3). Although most of our participants had reported sexual activity (74.9%) along with irregular condom use (81.5%), these behaviors were not associated with HBV exposure. Some additional factors, such as intravenous drug use, history of blood transfusion, and tattooing, were seldom referred by the participants of the study, thus precluding statistical analysis. Comparison between HBV epidemiological features among Afro-descendants in the past (1999–2003) and present (2015–2016) is shown in Fig. 2.

**Table 3:** Factors associated with hepatitis B virus exposure among Afro-descendant communities, Brazil Central, 2015–2016.

Factors	HBV prevalence	%	OR (95% CI)	*p*-value	Adjusted^c^ OR (95%CI)	*p*-value
***Community***						
SB	27/75	36.0	1		1	
FD	73/112	65.2	3.33 (1.81–6.13)	<0.001	4.22 (2.04–8.76)	<0.001
***Gender***						
Female	50/96	52.1	1			
Male	50/91	54.9	1.12 (0.63–1.99)	0.695	—	—
***Age (years****)*						
1–10	1/17	5.9	1		1	
11–20	6/22	27.3	6.00 (0.65–55.66)	0.115	8.94 (0.90–89.06)	0.062
21–30	8/11	72.7	42.67 (3.81–478.42)	0.002	59.91 (4.81–745.55)	0.001
31–40	16/32	50.0	16.00 (1.89–135.42)	0.011	22.07 (2.42–201.33)	0.006
41–50	27/43	62.8	27.00 (3.26–223.33)	0.002	29.76 (3.42–258.67)	0.002
51–60	19/31	61.3	25.33 (2.96-216.54)	0.003	37.82 (4.01–356.54)	0.002
> 60	23/31	74.2	46.00 (5.23–404.73)	0.001	63.63 (6.56–617.56)	<0.001
***Educational level (years)***						
≥10	18/33	54.6	1			
1–9	72/138	52.2	0.91 (0.42–1.95)	0.806	—	—
0	10/16	62.5	1.39 (0.41–4.72)	0.598	—	—
***History of surgery***						
No	51/96	53.1	1			
Yes	49/91	53.9	1.03 (0.58–1.83)	0.921	—	—
***History of a family member HBV-infected***^***a***^						
No	49/111	44.1	1			
Yes	41/64	64.1	2.26 (1.20–4.25)	0.012	2.79 (1.32–5.91)	0.007
***People per housing***^***a***^						
1–4	76/137	55.5	1			
≥5	24/49	49.0	0.77 (0.40–1.48)	0.434	—	—
***Shared personal objects***^***a***^						
No	56/108	51.9	1			
Yes	42/75	56.0	1.18 (0.65–2.14)	0.580	—	—
***History of blood contact***^***a***^						
No	93/170	54.7	1			
Yes	5/13	38.5	0.52 (0.16–1.65)	0.265	—	—
***Sexual activity***^**b**^						
No	6/12	50.0	1			
Yes	93/156	59.6	1.48 (0.46–4.78)	0.516	—	—
***Steady partner***^**b**^						
Yes	59/94	62.8	1			
No	40/74	54.1	0.70 (0.38–1.30)	0.255	—	—
***Condom use***^***b***^						
Always	9/18	50.0	1			
Occasionally/Never	82/134	61.2	1.58 (0.59–4.23)	0.366	—	—
***History of STI***^***b***^						
No	81/134	60.5	1			
Yes	8/14	57.1	0.87 (0.29–2.66)	0.810	—	—

95% CI: 95% confidence interval; OR: Odds ratio; STI: sexually transmitted infection. ^a^The total represents the number of individuals who answered the question. ^b^The total represents the number of individuals ≥13 years old who answered the question. ^c^Adjusted for community, age, and history of a family member HBV-infected.

### Hepatitis B virus vaccination like profile

Of the total 331 participants, 200 (60.4%) self-reported receiving HBV vaccine. However, only 93 (46.5%) of them had serological HBV vaccination like profile (isolate anti-HBs ≥10mIU/mL), 31.5% had evidence of exposure (total anti-HBc positive and HBsAg negative), and 22.0% were still susceptible to HBV infection. HBV vaccination like profile was found among 50.0% of those who reported receiving 3 HBV vaccine doses. Calculation of Ives–Gibbons correlation coefficient (r_264_ = 0) showed poor agreement between self-reported HBV vaccination and the number of persons with isolated anti-HBs (≥10mIU/mL). This finding is supported by poor sensitivity (0.79), specificity (0.27) and positive predictive value (0.46) of the self-reported answers.

The highest prevalence rate of HBV vaccination like profile occurred in the age group of 21–30 years. Thereafter, with increasing age, there was a decrease in this profile. By contrast, we observed an increasing anti-HBc positivity along with increasing age. After multiple linear regression analysis, being an FD community resident, belonging to the age group of 21–30 years and having an educational level ≥10 years were associated with the presence of HBV vaccination like profile. Individuals from SB community was almost three times greater odds for HBV susceptibility than that observed among FD residents (Table 4).

**Table 4:** Variables associated with HBV vaccination like profile among Afro-descendant communities, Brazil Central, 2015-2016.

Variables	Vaccinated like profile	%	OR (95% CI)	*p*-value	Adjusted^c^ OR (95%CI)	*p*-value
***Community***						
SB	49/97	50.5	1		1	
FD	95/134	70.9	2.38 (1.38–4.12)	0.002	2.89 (1.50–5.54)	0.001
***Gender***						
Female	94/140	67.1	1		1	
Male	50/91	54.9	0.60 (0.35–1.03)	0.062	0.67 (0.36–1.23)	0.197
***Age (years****)*						
1–10	26/42	61.9	1		1	
11–20	36/52	69.2	1.38 (0.59–3.26)	0.457	1.68 (0.66–4.30)	0.277
21–30	40/43	93.0	8.21 (2.17–30.97)	0.002	7.40 (1.78–30.71)	0.006
31–40	16/32	50.0	0.62 (0.24–1.56)	0.307	0.57 (0.19–1.70)	0.313
41–50	8/24	33.3	0.31 (0.11–0.88)	0.028	0.33 (0.11–1.04)	0.058
51–60	12/24	50.0	0.62 (0.22–1.70)	0.348	0.66 (0.22–1.97)	0.455
> 60	6/14	42.9	0.46 (0.14–1.58)	0.217	0.73 (0.19–2.76)	0.646
***Educational level (years)***						
≥10	48/63	76.2	1		1	
1–9	86/152	56.6	0.41 (0.21–0.79)	0.008	0.33 (0.14–0.78)	0.012
0	10/16	62.5	0.52 (0.16–1.67)	0.273	0.51 (0.12–2.05)	0.341
***History of a family member HBV-infected***^***a***^						
No	105/167	62.9	1			
Yes	31/54	57.4	0.80 (0.43–1.49)	0.473	—	—
***History of STI***^***b***^						
No	83/136	61.0	1			
Yes	10/16	62.5	1.06 (0.37–3.10)	0.909	—	—

95% CI: 95% confidence interval; OR: Odds ratio; STI: sexually transmitted infection. ^a^The total represents the number of individuals who answered the question. ^b^The total represents the number of individuals ≥ 13 years old who answered the question.^c^Adjusted for community, gender, age, and educatal level.

### Afro-descendants isolates belong to “Asian-American clade” of HBV/A1 subgenotype

**Figure 3:**
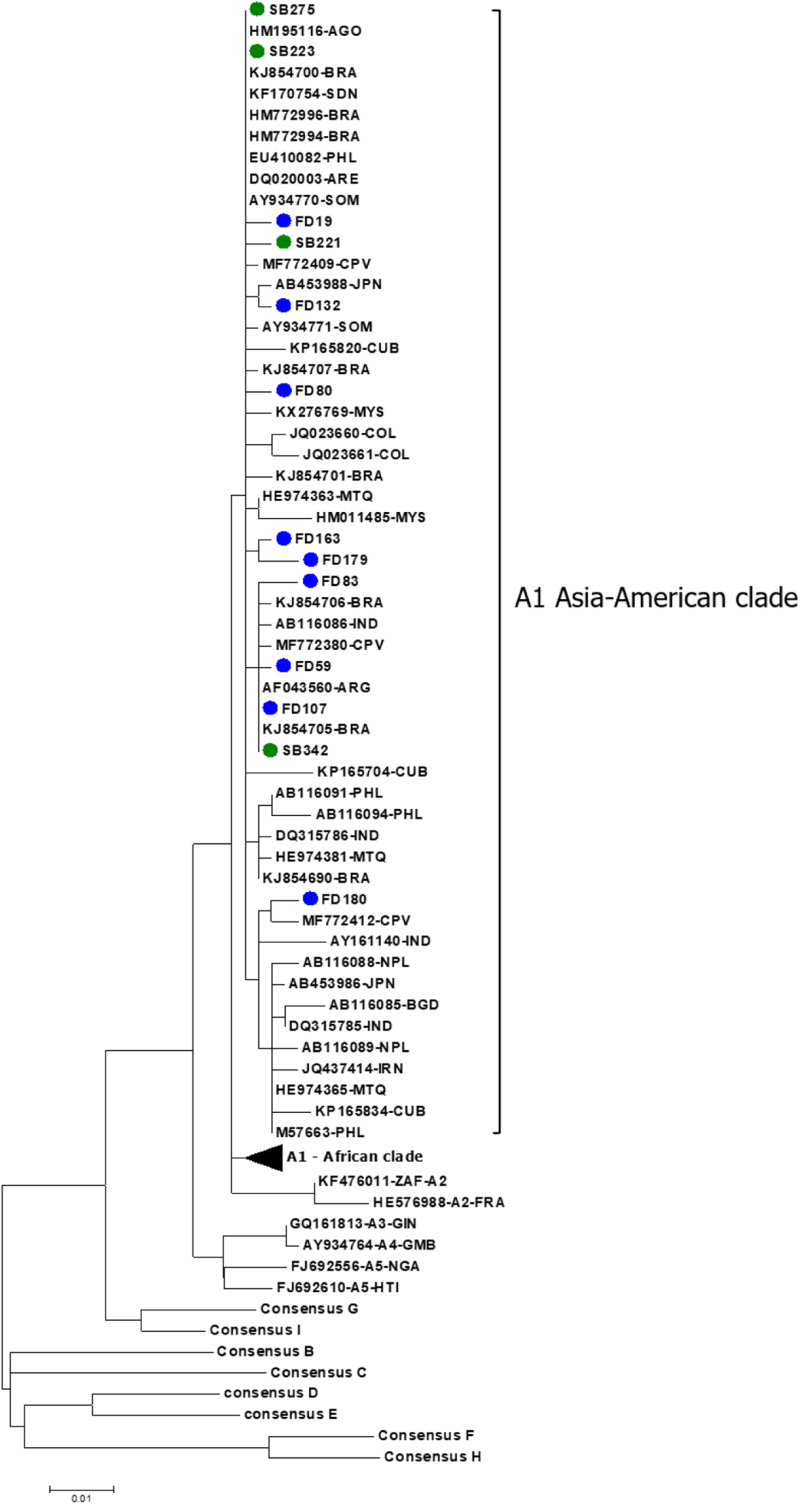
Phylogenetic tree analysis of the pre-S/S/Pol region of HBV genome, containing 60 HBV/A1, 6 HBV/A2-*quasi*A3 isolates obtained from GenBank database in addition to consensus sequences from HBV genotypes B, C, D, E, F, G, H and I. FD isolates are shown in blue filled circles and SB isolates are shown in green filled circle. The phylogenetic tree was performed using the Maximum Likelihood method and bootstrap resampling test with 1,000 replicates.

In this study, 13/18 (72.2%) HBsAg positive samples were successfully sequenced. Phylogenetic tree (Fig. 3) revealed that all 13 isolates belong to HBV subgenotype A1. Furthermore, all of them clustered with sequences previously classified into the HBV/A1 Asian-American clade^17^. Deduced amino acid of the viral surface protein and the overlapped polymerase reverse transcriptase domain demonstrated that none of these HBV isolates displayed drug resistance or vaccine escape mutations.

## Discussion

Information regarding trends in the epidemiology of hepatitis B is essential for improving public health, especially in remote and poor regions where a high prevalence of HBV infection was previously reported. In addition, population-based surveys in Brazil usually assess the prevalence rate of HBV exposure in a representative sample of the Brazilian State capitals^10,11^; thus, they may not reflect these difficult-to-access communities^7^.

In order to verify trends in hepatitis B epidemiological profile in two large remnant quilombo communities from Central Brazil, we reassessed Furnas dos Dionísios (rural) and São Benedito (urban) communities after over 13 years. Considering these two communities together, the prevalence rates of HBV exposure (30.2%) and hepatitis B chronic infection (5.4%) remained high, especially when compared to the general population from the same state (3.04% and 0.19%, respectively)^18^. These data vouch, once again, that Brazil has a heterogeneous HBV distribution despite being a low endemic country and the communities studied still represent foci of high prevalence of HBV infection^7,12,14^. The maintenance of chronic carries was expected, once HBV genotype A, known as having the highest risk of progression to chronicity^19^, was found in these populations. Moreover, the possibility of vertical transmission of HBV in these communities is also related to HBV chronicity^4,6,20,21^.

In our study, one individual aged <10 years was found to be anti-HBc/anti-HBs positive. His mother is a chronic carrier of HBV infection, which may justify his exposure to HBV at such a young age. The other two individuals aged 13 years (without sexual activity) were also found to be exposed to HBV infection. One of them was anti-HBc positive and has an HBV chronic carrier mother. The other one is anti-HBc/anti-HBs positive and has both parents exposed to HBV infection.

In 2003, our research group conducted actions for health promotion such as HBV vaccination and health education campaigns in remnant quilombo communities, including SB and FD. Low compliance with the full vaccination scheme (28%) and low response to HBV vaccination (83.1%) were observed^15^. In view of this, we conducted house-to-house visits in the FD communities, offering HBV immunization once again, raising awareness, and engaging families to increase HBV vaccine adherence, especially among children and young people.

Over 13 years after preventive interventions, a significant increase in the proportion of individuals with serological HBV vaccination like profile (10.7% to 43.5%; p < 0.001) and a significant decrease in the proportion of individuals susceptible to HBV infection (58.9% to 26.3%; p < 0.001) were observed in FD and SB communities (Fig. 2). In addition, the highest rate of HBV vaccination like profile (93.0%) observed among young adults aged 21–30 years probably reflects vaccination at childhood/adolescence during the campaigns promoted since 2003.

When separately analyzed, FD community, despite being a rural community, has a lower proportion of individuals who are still susceptible to HBV infection compared to SB (18.8% vs. 38.7%, respectively; p < 0.001), however, it has a higher prevalence of HBV exposure (Table 2). In the last 13 years, the significant improvement in the proportion of individuals with serological HBV vaccination like profile observed in FD (2.0% to 45.9%; p < 0.001) was probably due to the house-to-house HBV immunization and to the engagement of a health worker, who is a member of the community, working on health education. Since the FD community area is not extensive, the cost-effectiveness of offering HBV vaccine and health education in the residences seems to be worthwhile.

Our findings, most likely a consequence of the health promotion campaigns that were offered in the past, reinforce the importance of expanding and maintaining hepatitis B immunization strategies in order to increase coverage for all children, high-risk populations and vulnerable groups such as Afro-Brazilian communities to achieve the goal set by the United Nations in 2015 of eliminating HBV infection as a public health threat by 2030^22,23^.

Compared with data from 2003 and 2005^12,14^, considering 1,058 Afro-descendants living in twelve communities from Mato Grosso do Sul, Brazil Central, this study demonstrated that increasing age and history of a family member HBV-infected remain to be important independent factors associated with HBV exposure. This is in line with many epidemiological studies conducted in different group populations. The association between HBV exposure and increasing age could be explained by the years of exposure to HBV over a lifetime, mainly among those who reported a history of a family member HBV-infected^2,24–26^.

Once a considerable number of HBV infections are due to perinatal or early childhood transmission, ensuring HBV vaccination to all newborns, even those born at home, is an additional challenge. Association between being a resident of FD community and a high prevalence of HBV exposure observed in this study was expected since the FD community has a higher prevalence rate of HBV exposure and HBV chronic carriers. Missed opportunity for infant vaccination before our first intervention, particularly among those born at home, may also explain the high prevalence of HBV exposure found in FD community (rural), especially in people with increased ages. Such a scenario has been documented in several countries of sub-Saharan Africa, from where Africans slaves came to Brazil during the slave period. It is possible that the viral dynamics in these quilombo communities, where the flow of people is reduced, still reflects the high endemicity and genetic variability of the original African ancestors^12,14,27^.

Phylogenetic analysis revealed that all thirteen samples clustered in the called “Asian-American” clade from subgenotype A1, the genotype most prevalent (86%) in FD community in the past (Fig. 3)^20^. The “Asian-American” or “Cosmopolitan” clade comprises isolates from Asia, Somalia, Angola, Cape Verde, Caribbean islands, which also received African workforce in the past, and a restricted number of South American strains^17,28–30^. This clade would have originated in the nineteenth century when African slaves were forced-exported from East Africa to Asia and Central and South America^27^. HBV subgenotype A1 has also been described in other Afro-descendant communities from Brazil, in the states of Maranhão and Goiás^11,24^. Once Angola was the main supplier of African slaves to Brazil from 16 to 18^th^ centuries^27^, the clustering of an Angolan sequence with afro-Brazilian isolates in the “Asian-American” clade may provide evidence of the circulation of genetically related viral isolates in this country during the slave trade period. Despite the African subgenotype D4 has been detected in rural populations with African ancestry in North and Northeast Brazil^31,32^, no D4 sequence has been identified in this study. The absence of this African subgenotype in Afro-Brazilian communities in Central Brazil may be due to differences in the colonization process along the Brazilian territory (captives from different African regions at different historical times), leading the northeastern states to have an HBV genotypic distribution similar to North America and the Caribbean countries^31,33,34^.

Amino acid analysis of the viral polymerase reverse transcriptase domain demonstrated that none of the individuals presented drug resistance mutations. To our knowledge, none of the chronic carriers from this work received previous antiviral therapy, thus based on this genome fragment analysis, no primary resistance mutations have been introduced in FD and SB communities. Moreover, despite the low response to HBV vaccination documented in the past study^15^, no scape vaccine mutation was observed in the HBV surface gene in this work.

This work highlights the importance of encouraging HBV epidemiological studies in vulnerable, difficult-to-access and high-risk populations, once the preventive measures taken in the past have brought improvements in prevention and control of HBV infection. Even more important than only reassess these populations to verify the impact of the health interventions previously conducted, it is to plan for focused action and help public policy to prioritize the allocation of resources. A catch-up immunization with an alternative (accelerated) hepatitis B vaccine schedule along with the continued engagement of a community health worker acting on the awareness of the population about the importance of preventive measures, may be more effective to achieve high vaccination coverage in these hard-to-reach communities^35^.

Our data warn us that communities must be continuously made aware of the HBV vaccination importance; especially at birth, and also that they still lack appropriate access to vaccination and treatment. These findings serve as a tool to help us design better approach strategies to combat hepatitis B and eliminate this infection as a public health concern by 2030, especially in populations that are most affected, such as Afro-descendant communities. In conclusion, although the level of chronic hepatitis B infection was still high in 2015–2016 compared to 1999–2003, a significant increase in the prevalence rate of individuals with serological HBV vaccination like profile and a decrease of susceptible individuals to HBV infection in FD and SB communities were observed. These findings reinforce the importance of promoting HBV vaccination to all vulnerable and difficult-to-access populations to close gaps in prevention. Further epidemiological studies in Afro-descendant communities are needed for a continuous hepatitis B monitoring in order to evaluate the true burden of HBV infection, specific risk factors, and targeted prevention measures in the next decade.

## Methods

### Ethics statement

Informed written consent was obtained, after a detailed explanation of the study, at the time of sampling from all participants or their legal guardians, in case of individuals under age 18 or with a mental disability. Post-test counseling was offered to all participants. All participants who tested positive for HBsAg were referred to public health units for access to treatment. The study protocol was approved by the Ethics Committee of the Federal University of Mato Grosso do Sul (CAAE 35103214.0.0000.0021 and 1.210.184). All methods were performed in accordance with the relevant guidelines and regulations established by the Ethics Committee.

### Study subjects

Between October 2015 and July 2016, residents of two remnant quilombo communities in the state of Mato Grosso do Sul, Central Brazil, were invited to participate in this cross-sectional survey. Furnas do Dionísio (FD), a rural community with approximately 450 residents located in the city of Jaraguari, 45 km from the capital Campo Grande and São Benedito (SB), an urban community located in the capital with about 300 residents. Based on an estimated prevalence of HBV exposure of 19.8%^12^, a significance level of 95% (α < 0·05), an accuracy of 5% and a design effect of 1.0, sample size calculation yielded 244 individuals. We included 20% more individuals (total of 292) to account for anticipated loss due to refusal to participate. Considering the prevalence of HBV exposure in Furnas dos Dionísio (42.4%) and São Benedito (16.1%), the minimum required sample stratified by community was 205 and 123, respectively. Blood samples were collected from 331 Afro-descendants, 207 from FD and 124 from SB, previously interviewed regarding their socioeconomic and behavioral characteristics as well as their knowledge about prior HBV vaccination.

### Serological tests

Serum samples were screened by enzyme-linked immunosorbent assay (ELISA) for the presence of HBsAg, hepatitis B core antibody (total anti-HBc), and hepatitis B surface antibody (anti-HBs) (Biokit S. A., Barcelona, Spain). Cobas® e601 Analyzer (Roche Diagnostics, Mannheim, Germany) was used to test the presence of hepatitis B e antigen (HBeAg), antibodies against HBeAg (anti-HBe) and anti-HBc IgM in HBsAg-positive samples. Current HBV infection was defined as a positive HBsAg test result. HBV exposure was defined as a positive anti-HBc and/or HBsAg test result. HBV vaccination like profile included all participants with the anti-HBs positive result (≥10mIU/mL) combined with negative anti-HBc and HBsAg results.

### Viral nucleic acid extraction

Nucleic acid (DNA) was extracted from 200 μL sera of HBsAg positive samples using the High Pure Viral Nucleic Acid kit (Roche Diagnostics, Mannheim, Germany) following the manufacturer’s instruction and aliquots were stored at −70 °C until use.

### HBV-DNA amplification and purification

The complete pre-S/S HBV region (nt 2826 to nt 841) was amplified by semi-nested polymerase chain reaction (PCR). The first round of amplification was performed with 3 µL of DNA and 0.4 µL of Taq DNA polymerase 5U/µL (Amplitaq, Applied Biosystems, Foster City, CA) in a final volume of 25 µL using the following cycling condition: 94 °C for 2 min (denaturation step), 30 cycles at 94 °C for 30 s, 52 °C for 30 s and 72 °C for 90 s, followed by a final extension of 72 °C for 7 min.

The second round of amplification was performed with 2 µL of the PCR product from the first round and 0.5 µL of Taq DNA polymerase 5U/µL in a final volume of 50 µL using the following cycling condition: 94 °C for 3 min (denaturation step), 30 cycles at 95 °C for 30 s, 55 °C for 40 s and 72 °C for 2 min, followed by a final extension of 72 °C for 7 min. PCR products were loaded onto 1.5% agarose gels, separated by electrophoresis, stained with ethidium bromide and visualized under UV light. Agarose gel bands containing DNA fragments sizing ~1200 bp were then purified using the illustra™ GFX™ PCR DNA and Gel Band Purification Kit (GE Healthcare Bio-Sciences Corp., NJ 08855–1327, USA) according to the manufacturer’s instruction.

### Sequencing and phylogenetic analyses

Nucleotide sequences of pre-S/S region was determined by direct sequencing using the BigDye Terminator v3.1 Cycle Sequencing kit (Applied Biosystems, Foster City, CA). Sequencing reactions were analyzed on an ABI 3730 automated sequencer (Applied Biosystems). Multiple sequence alignment was performed by Clustal X program. The dataset was composed by 74 sequences representative of all HBV genotypes (consensus sequences, n = 8), HBV/A subgenotypes A2 and quasi-A3 (n = 6) and HBV/A1 sequences (n = 60) representative of both African and Asia-American clades. Phylogenetic analysis was performed by the Maximum Likelihood method (bootstrap resampling test with 1,000 replicates), implemented in MEGA version 7 software, under the general time reversible with gamma distributed rate heterogeneity and estimated proportion of invariable sites (GTR + G + I) substitution model, which was selected as the best-fit model. All sequences employed in the phylogenetic analysis are listed in supplementary table.

### Statistical analysis

Data analysis was performed in the statistical software Stata SE, version 13 (StataCorp LP, College Station, USA). This study used the chi-square test (χ^2^) or the Fisher’s test (for categorical variables) to assess differences between proportions and determine *p* values (two-tailed). The prevalence rate of HBV exposure and a 95% confidence interval (CI) were calculated. Odds ratios and 95% confidence intervals (CI) were used to verify potential predictors of HBV infection and/or exposure (presence of anti-HBc and/or HBsAg markers). Variables presenting a p-value <0·20 were included in multiple linear regression analysis. The selection of variables for the final model was performed stepwise, according to the number of events per variable (EPV). Hosmer-Lemeshow test was used to assess goodness-of-fit, choosing the best regression equation^36^. P-values <0·05 were considered statistically significant. Individuals with anti-HBs alone (≥10 mIU/mL) were excluded from the HBV associated factors analysis since it probably indicates an HBV vaccination like profile (n = 187). Sensitivity, specificity and positive predictive value (PPV) were calculated to verify the reliability of HBV vaccination self-report and serologic test results were used as “gold standard” indicators of HBV vaccination like profile. Ives–Gibbons correlation coefficient calculation was performed to verify the agreement between HBV vaccination self-report and the number of individuals presenting anti-HBs alone^37^.

## Limitations

This work is a cross-section study with convenience sampling and as such, our sample size might not represent the population as a whole and be biased by volunteers. Self-reporting, recall-bias, as well as lack of vaccination records, are some of the limitations of this study. Vaccination card was not available for verification, due to the special circumstances under which this study was conducted. Besides, once some HBV vaccinated individuals lose detectable levels of anti-HBs over time, the frequency of susceptibility may be overestimated.

## Supplementary information

Supplementary table.
