# Author Correction: Hepatitis B: changes in epidemiological features of Afro-descendant communities in Central Brazil

**DOI:** 10.1038/s41598-020-68528-8

**Published:** 2020-07-07

**Authors:** Livia Alves Lima, Bárbara Vieira do Lago, Sabrina Moreira dos Santos Weis-Torres, Regina Maria Bringel Martins, Gabriela Alves Cesar, Larissa Melo Bandeira, Grazielli Rocha Rezende, Andrea de Siqueira Campos Lindenberg, Selma Andrade Gomes, Ana Rita Coimbra Motta-Castro

**Affiliations:** 1 Federal University of Mato Grosso Do Sul, Campo Grande, MS Brazil; 2 Oswaldo Cruz Foundation, FIOCRUZ, Rio de Janeiro, RJ Brazil; 3 Institute of Technology in Immunobiology, Bio-Manguinhos, FIOCRUZ, Rio de Janeiro, RJ Brazil; 4 Federal University of Goiás, Goiânia, GO Brazil; 5 Oswaldo Cruz Foundation, FIOCRUZ Mato Grosso Do Sul, Ministry of Health, Campo Grande, MS Brazil

Correction to: *Scientific Reports* https://doi.org/10.1038/s41598-020-63094-5, published online 21 April 2020.

The Acknowledgements section in this Article is incomplete.

“We would like to thank Dr. Christian Niel for his valuable comments during the development of this study. Also, we would like to thank all the health professionals who supported the first steps of this project, especially Dr. Marco Puga and Tayana Ortiz Tanaka. This work was supported by the Federal University of Mato Grosso do Sul - UFMS/MEC - Brazil, National Council for Scientific and Technological Development (CNPq), Foundation for Support to the Development of Education, Science and Technology of the State of Mato Grosso do Sul (FUNDECT) and the Coordination for the Improvement of Higher Education Personnel (CAPES).”

should read:

“We would like to thank Dr. Christian Niel for his valuable comments during the development of this study. Also, we would like to thank all the health professionals who supported the first steps of this project, especially Dr. Marco Puga and Tayana Ortiz Tanaka. This work was supported by the Federal University of Mato Grosso do Sul - UFMS/MEC - Brazil, National Council for Scientific and Technological Development (CNPq), Foundation for Support to the Development of Education, Science and Technology of the State of Mato Grosso do Sul (FUNDECT) and the Coordination for the Improvement of Higher Education Personnel (CAPES). This study was financed in part by the Coordenação de Aperfeiçoamento de Pessoal de Nível Superior – Brasil (CAPES) – Finance Code 001.”

