# Supplementary material for: Hepatitis B: changes in epidemiological features of Afro-descendant communities in Central Brazil
Source: Sci Rep. 2020 Apr 21;10:6708. doi: 10.1038/s41598-020-63094-5 (PMC7174315; doi:10.1038/s41598-020-63094-5)
Supplement: Supplementary file 1 — Supplementary table. [file 41598_2020_63094_MOESM1_ESM.docx]

Hepatitis B: changes in epidemiological features of Afro-descendant communities in Central Brazil

Short title: Hepatitis B in Afro-Brazilian communities

Livia Alves Lima^1^, Bárbara Vieira do Lago^2,3*^, Sabrina Moreira dos Santos Weis-Torres^1^, Regina Maria Bringel Martins^4^, Gabriela Alves Cesar^1^, Larissa Melo Bandeira^1^, Grazielli Rocha Rezende^1^, Andrea de Siqueira Campos Lindenberg^1^, Selma A. Gomes^2^, Ana Rita Coimbra Motta-Castro^1,5*^

^1^Federal University of Mato Grosso do Sul, Campo Grande, MS, Brazil;

^2^Oswaldo Cruz Foundation, FIOCRUZ, Rio de Janeiro, RJ, Brazil;

^3^Institute of Technology in Immunobiology, Bio-Manguinhos, FIOCRUZ, Rio de Janeiro, RJ, Brazil;

^4^Federal University of Goiás, Goiânia, GO, Brazil;

^5^Oswaldo Cruz Foundation, FIOCRUZ, Campo Grande, MS, Brazil.

Correspondence:[*barbaravlago@gmail.com](mailto:*barbaravlago@gmail.com); *arcm.castro@hotmail.com

| **Accession number** | **Location** | **Subgenotype** |
| --- | --- | --- |
| AB076678 | Malawi | A1 |
| AB076679 | Malawi | A1 |
| AB116085 | Bangladesh | A1 |
| AB116086 | India | A1 |
| AB116088 | Nepal | A1 |
| AB116089 | Nepal | A1 |
| AB116091 | Phillippines | A1 |
| AB116094 | Phillippines | A1 |
| AB453986 | Japan | A1 |
| AB453988 | Japan | A1 |
| AF043560 | Argentina | A1 |
| AF297623 | South Africa | A1 |
| AY161140 | India | A1 |
| AY233274 | South Africa | A1 |
| AY233275 | South Africa | A1 |
| AY934764 | Gambia | A4 (*quasi*-A3) |
| AY934770 | Somalia | A1 |
| AY934771 | Somalia | A1 |
| AY934772 | Uganda | A1 |
| AY934773 | Tanzania | A1 |
| DQ020002 | Congo | A1 |
| DQ020003 | United Arab Emirates | A1 |
| DQ315785 | India | A1 |
| DQ315786 | India | A1 |
| EU185789 | Argentina | A1 |
| EU410082 | Phillippines | A1 |
| FJ692556 | Nigeria | A5 (quasi-A3) |
| FJ692571 | Haiti | A1 |
| FJ692610 | Haiti | A5 (quasi-A3) |
| FM199977 | Rwanda | A1 |
| GQ161813 | Guinea | A3 (quasi-A3) |
| HE576988 | France | A2 |
| HE974363 | Martinique | A1 |
| HE974365 | Martinique | A1 |
| HE974381 | Martinique | A1 |
| HM011485 | Malaysia | A1 |
| HM1951116 | Angola | A1 |
| HM535205 | Zimbabwe | A1 |
| HM772994 | Brazil | A1 |
| HM772996 | Brazil | A1 |
| JN182319 | South Africa | A1 |
| JQ023660 | Colombia | A1 |
| JQ023661 | Colombia | A1 |
| JQ437414 | Iran | A1 |
| JX154579 | Kenya | A1 |
| KF170754 | Sudan | A1 |
| KF476011 | South Africa | A2 |
| KF922434 | South Africa | A1 |
| KJ854690 | Brazil | A1 |
| KJ854700 | Brazil | A1 |
| KJ854701 | Brazil | A1 |
| KJ854705 | Brazil | A1 |
| KJ854706 | Brazil | A1 |
| KJ854707 | Brazil | A1 |
| KP165704 | Cuba | A1 |
| KP165820 | Cuba | A1 |
| KP165834 | Cuba | A1 |
| KX276769 | Malaysia | A1 |
| KX982157 | Rwanda | A1 |
| M57663 | Phillippines | A1 |
| MF615980 | Mozambique | A1 |
| MF772380 | Cape Verde | A1 |
| MF772409 | Cape Verde | A1 |
| MF772412 | Cape Verde | A1 |

**Supplementary table:** HBV sequences used to construct phylogenetic tree. Sequences are identified by their GenBank accession numbers, countries of origin and HBV subgenotype classification.
